# Effects of NaCl and CaCl_2_ as Eustress Factors on Growth, Yield, and Mineral Composition of Hydroponically Grown *Valerianella locusta*

**DOI:** 10.3390/plants12071454

**Published:** 2023-03-26

**Authors:** Orfeas Voutsinos-Frantzis, Ioannis Karavidas, Dimitrios Petropoulos, Georgios Zioviris, Dimitrios Fortis, Theodora Ntanasi, Andreas Ropokis, Anestis Karkanis, Leo Sabatino, Dimitrios Savvas, Georgia Ntatsi

**Affiliations:** 1 Laboratory of Vegetable Production, Department of Crop Science, Agricultural University of Athens, 11855 Athens, Greece; 2 Department of Agriculture Crop Production and Rural Environment, University of Thessaly, 38446 Volos, Greece; 3 Department of Agricultural, Food and Forest Sciences (SAAF), University of Palermo, Viale delle Scienze, Ed. 5, 90128 Palermo, Italy

**Keywords:** corn salad, salt stress, hydroponics, nitrates, essential elements, isosmotic

## Abstract

Corn salad (*Valerianella locusta*) is a popular winter salad, cultivated as an ingredient for ready-to-eat salads. The application of mild salinity stress (eustress) can increase the flavor and reduce the nitrate content of certain crops but, at the same time, a wrong choice of the eustress type and dose can negatively affect the overall productivity. In this research, the effects of different isosmotic salt solutions, corresponding to two different electrical conductivity (EC) levels, were investigated on the yield and mineral composition of hydroponically grown *Valerianella locusta* “Elixir”. Five nutrient solutions (NS) were compared, including a basic NS used as the control, and four saline NS were obtained by adding to the basic NS either NaCl or CaCl_2_ at two rates each, corresponding to two isosmotic salt levels at a low and high EC level. Corn salad proved moderately susceptible to long-term salinity stress, suffering growth losses at both low and high EC levels of saline solution, except from the low NaCl treatment. Hence, it appears that mild salinity stress induced by NaCl could be employed as an eustress solution and corn salad could be cultivated with low-quality irrigation water (20 mM NaCl) in hydroponic systems.

## 1. Introduction

In the last decade, the share market of minimally processed vegetables has increased significantly, placing them among the fastest-growing consumed foods worldwide [1]. Consumers have emphasized the need for higher quality and tastier ready-to-eat vegetables in their effort to diversify a monotonous diet and consume healthier foods [2,3,4]. Ready-to-eat vegetables are becoming more and more common thanks to the advantages they provide to the end-user, such as reducing the preparation time and on-site waste [5]. Nevertheless, the complex industrial processes involved in producing these salads can have certain environmental impacts, including the consumption of water for washing and the generation of plastic waste [6]. Moreover, the decrease in essential minerals, chemicals, and bioactive compounds found in fresh-cut salads can directly impact consumers by reducing the nutrient content of their diet [7,8,9].

*Valerianella locusta* L., also known as corn salad or lamb’s lettuce, is a leafy vegetable commonly used nowadays in fresh-cut salads and salad mixes [10]. Its introduction into Western European agriculture dates back to the 17th century, with France being one of the early adopters [11]. In 1998, France was responsible for 75% of the world’s corn salad production, and 90% of that came from the region of Nantes. In 2004, corn salad was considered the third-most important greenhouse-grown crop in Germany, after tomato and cucumber [12]. The same trend is also present in Italy, where the revaluation of the Mediterranean diet and the need for high-quality and nutritious fresh-cut salads has also increased the interest for corn salad production under protected cultivation or open fields [13].

Harvesting can take place at different stages depending on the purpose of the cultivation. For fresh-cut salads sealed in plastic bags, corn salad can be harvested as soon as 3–4 pairs of leaves are developed (known as the *4ème gamme* in France). When the produce is sold in plastic punnets, 4–6 pairs of leaves are needed, and for traditional sale in trays, each plant should have 7–8 pairs of leaves [11]. The commercial interest for corn salad has increased due to its leaves’ mild and delicate taste, and overall dietary characteristics and rich bioactive compound content [14,15]. Towards that end, the rosettes are known to contain several bioactive compounds, such as vitamin C, carotenoids, phenols, folic acid, sterols, and omega-3 fatty acids [16,17,18,19]. This commercial interest has encouraged producers around Europe to grow lamb’s lettuce on different scales, large or small, either in greenhouses or in open fields, and sell their produce to supermarkets and delicatessen restaurant supply chains [9]. Moreover, corn salad’s market demand has been discussed in popular agro-websites such as Hortidaily [20] and Fresh Plaza [21].

As a plant, corn salad is an annual leafy vegetable of Mediterranean origin. This wild species was found growing in fallow land and cultivated fields, as a weed, during the winter season [11]. Corn salad is tolerant to low temperatures and the canopy can even survive under snow cover [9,22,23,24]. On the other hand, it has been observed that in hydroponically grown corn salad, root temperature can negatively affect the plant when it surpasses 25 °C [25,26]. As they grow, the seedlings develop into a rosette presenting six-to-seven pairs of opposite leaves that grow at right angles to the previous pair forming crosses [11]. In terms of nutritional and agronomical characteristics, depending on the cultivation method, yield, nitrate levels, and the quality of corn salad can differ [27]. Towards that end, several concerns have been raised in recent decades regarding the nitrate content of vegetables [28,29,30,31,32].

Groundwater salinization due to the presence of salts such as NaCl and CaCl_2_ is a growing concern in several regions of the world, including coastal, arid, and semi-arid areas such as those of the Mediterranean basin, Iran, India, Egypt, South Africa, Pakistan, and Saudi Arabia [33,34,35,36,37,38,39,40]. These salts can come from various sources such as seawater intrusion, dissolution of minerals in rocks, and anthropogenic activities [41]. Towards that end, soilless culture has been considered a solution for cultivating plants in arid and semi-arid regions, where water scarcity, soil salinity, and soil health are a problem [42,43,44]. Waters with comparable NaCl and CaCl_2_ concentrations to those used in the study can be found in many of these regions where saline irrigation water is commonly used.

The need for better control of vegetable production (yield, nitrate levels, etc.) has led to the adoption of soilless culture in commercial horticulture [45,46]. Closed-loop hydroponics have also gained more and more interest due to their water saving attributes and environmentally protective nature, since they avoid nutrient leaching [47,48]. From all the different soilless culture methods, the floating irrigation system (FL) has been widely applied to the cultivation of leafy vegetables such as rocket, corn salad, lettuce, spinach, and certain herbs, thanks to its low maintenance needs, relatively low labor costs, high water use efficiency, and high greenhouse space use efficiency [49]. Furthermore, FL allows for high sanitary quality and the harvesting of clean materials, thus minimizing the need for thorough washing treatments for vegetables that are cultivated for fresh-cut salads [50]. In addition, the nitrate content can be manipulated to a certain degree through the nutrient solution and be kept under certain thresholds [51,52,53,54,55,56]. Depending on the available technology of the greenhouse, the nitrate content can be further controlled through supplemental lighting [57,58,59,60,61], but other parameters such as the growing period, temperatures, and time of harvest should also be taken into account since they affect the nitrate accumulation in leaf tissues [62,63].

This study focused on the effects of four salinity treatments, compared to a standard nutrient solution, on the growth and mineral composition of hydroponically grown *Valerianella locusta* “Elixir”, with the goal of reducing nitrate levels in the plant tissue. A control (Control) nutrient solution (NS), with osmotic potential −0.08 MPa, was compared to four eustress treatments. Each eustress treatment induced stress through the use of different levels of either NaCl or CaCl_2_. Moreover, the four eustress treatments were separated into two groups based on their electrical conductivity (EC) and osmotic potential. The osmotic potential of the low and high EC treatments was −0.18 and −0.28 (MPa), respectively. Therefore, the low EC NaCl (LNa) treatment was isosmotic to the low EC CaCl_2_ (LCa) treatment and the high EC NaCl (HNa) was isosmotic to the high EC CaCl_2_ (HCa) treatment.

## 2. Results

### 2.1. Growth and Productivity Response

**Figure 1:**
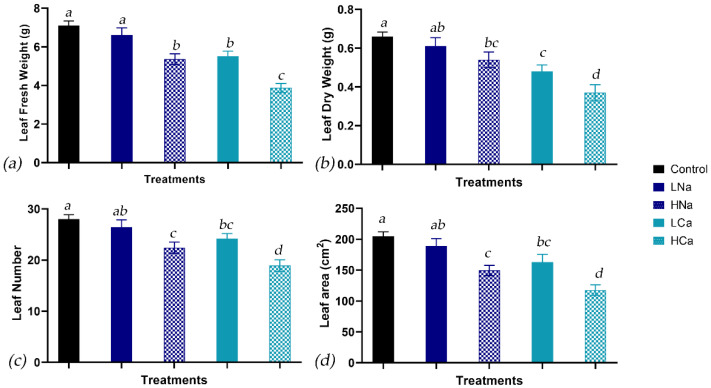
(**a**) Leaf fresh weight (LFW), (**b**) leaf dry weight (LDW), (**c**) leaf number (LN), and (**d**) leaf area (LA) in *Valerianella locusta* plants grown in a floating system, as influenced by four salinity treatments (referred to as low and high salinity level) and the salinity source. Control: standard nutrient solution (−0.08 MPa, 20 °C); LNa: low NaCl-salinity (−0.18 MPa, 20 °C); HNa: high NaCl-salinity (−0.28 MPa, 20 °C); LCa: low CaCl_2_-salinity (−0.018 MPa, 20 °C); HCa: high CaCl_2_-salinity (−0.28 MPa, 20 °C). Vertical bars indicate ± standard errors of means of three measurements. Same letters indicate non-significant differences at *p* ≤ 0.05 according to Duncan’s multiple range test.

To assess the effects on the growth and yield of corn salad, leaf fresh and dry weight (LFW and LDW), as well as leaf number (LN) and leaf area (LA), were measured at the harvest stage. As seen in Figure 1a, the leaf fresh weight (LFW) of LNa, LCa, HNa, and HCa was decreased by 7%, 21%, 25%, and 46%, respectively, compared to the control. In addition, the reduction of the leaf dry weight (LDW), as seen in Figure 1b, followed a similar trend. The highest values were observed under control conditions, and the LDW of LNa, LCa, HNa, and HCa were decreased by 8%, 27%, 18%, and 44%, respectively, compared to the control. In addition, leaf number (LN) and leaf area (LA) followed the same reduction in relation to the treatments. As seen in Figure 1c, the LN of LNa, LCa, HNa, and HCa were decreased by 6%, 14%, 20%, and 32%, respectively, compared to the control. Similarly, the LA of LNa, LCa, HNa, and HCa were decreased by 8%, 20%, 27%, and 43%, respectively, compared to the control.

**Figure 2:**
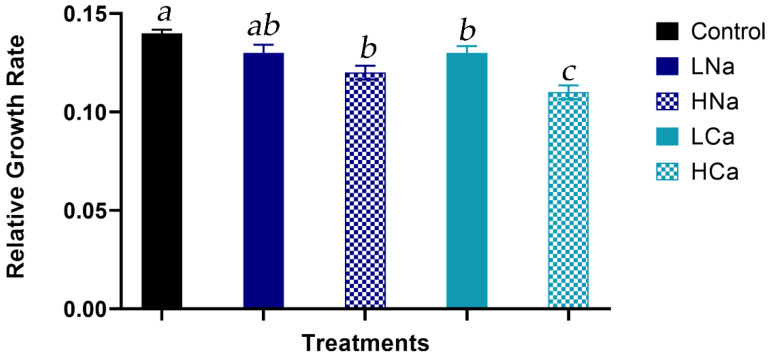
Relative Growth Rate (RGR) in *Valerianella locusta* plants grown in a floating system, as influenced by four salinity treatments (referred to as low and high salinity level) and the salinity source. Control: standard nutrient solution (−0.08 MPa, 20 °C); LNa: low NaCl-salinity (−0.18 MPa, 20 °C); HNa: high NaCl-salinity (−0.28 MPa, 20 °C); LCa: low CaCl_2_-salinity (−0.018 MPa, 20 °C); HCa: high CaCl_2_-salinity (−0.28 MPa, 20 °C). Vertical bars indicate ± standard errors of means of three measurements. Same letters indicate non-significant differences at *p* ≤ 0.05 according to Duncan’s multiple range test.

Using the data from Figure 1, the relative growth rate (RGR), expressed in g day^−1^, was calculated as defined in Section 4.3. As seen in Figure 2, the highest values were observed under control conditions, followed by LNa which was lower by 4% compared to the control and did not differ significantly from it. Moreover, the RGR observed under LNa conditions was similar to those of HNa and LCa, which were decreased by 9% and 10%, respectively, compared to the control. The RGR of plants grown under HCa conditions was 18% lower than the control and was the lowest compared to the rest treatments. 

**Figure 3:**
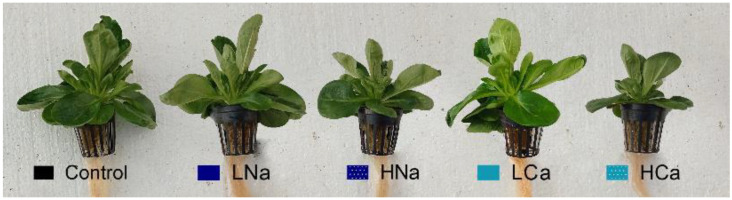
*Valerianella locusta* plants grown in a floating system at harvest stage, as influenced by four salinity treatments (referred to as low and high salinity level) and the salinity source. Control: standard nutrient solution (−0.08 MPa, 20 °C); LNa: low NaCl-salinity (−0.18 MPa, 20 °C); HNa: high NaCl-salinity (−0.28 MPa, 20 °C); LCa: low CaCl_2_-salinity (−0.018 MPa, 20 °C); HCa: high CaCl_2_-salinity (−0.28 MPa, 20 °C).

These results can also be visualized in Figure 3, where corn salad plants were placed next to each other for better comparison.

### 2.2. Nutritional Content of Leaf Tissues

#### 2.2.1. Nitrates and Total Kjeldahl Nitrogen

As far as the nitrate content is concerned (Table 1), the highest value was observed in the control, followed by LCa and LNa, with the latter having similar values to HNa which, however, had significantly less nitrate compared to the LCa. Moreover, the nitrate content of HNa was similar to that of HCa, which was the treatment with the lowest values compared to all the other treatments. The total Kjeldahl Nitrogen (Total-N) did not differ significantly between any of the treatments.

#### 2.2.2. Non-Treatment-Related Macronutrients (Mg, K, P) and Micronutrients (Cu, Zn, Fe, Mn, B)

Apart from nitrogen, the effect of the different isosmotic salt solutions and two EC levels on the concentration of other macro and micronutrients in the leaf tissues of *Valerianella locusta* was also investigated. As seen in Table 2, no variations were observed in the Mg, K, and P content of the plant leaf tissues regardless of the treatment applied.

Furthermore, as observed in Table 3, the only micro-element whose content was significantly affected by the different eustress treatments was boron. For the rest (Cu, Zn, Fe, and Mn), no significant differences were observed in the leaves. The LCa treatment exhibited the highest boron content in the leaf tissue. The HCa, LNa, and control treatments did not differ significantly from each other and were not significantly lower compared to LCa or significantly greater compared to HNa. The HNa treatment had significantly lower boron content compared to LCa. It is useful to note that even though zinc levels were lower than 200 mg Kg^−1^ of DW, which is a threshold that when exceeded can lead to phytoxicity [64], all treatments had accumulated zinc greater than 100 mg Kg^−1^ of DW. It is often referenced that the maximum threshold for zinc in vegetables is 99.4 mg of Zn per kg of dry weight [65,66,67,68]. These research articles usually refer to findings from evaluations conducted on zinc, in 1966 and 1982 [69]. However, it is important to consider that clinical studies have been used to establish the provisional maximum tolerable daily intake (PMTDI) of zinc, which stands at 0.3–1 mg kg^−1^ bw. These studies administered up to 600 mg of zinc sulfate, equivalent to 200 mg of elemental zinc, daily for several months, and no adverse effects were reported on blood counts and serum biochemistry. Furthermore, in 2003, WHO proposed that a guideline value for zinc was not needed based on recent human studies [70]. Further studies are needed to fully understand the potential health implications of consuming vegetables with elevated levels of zinc.

#### 2.2.3. Treatment-Related Nutrients and Non-Nutrients (Ca, Na, and Cl)

As a means to understand and differentiate the effects of osmotic stress from ion toxicity that might have resulted from the NaCl and CaCl_2_ “eustress” treatments, the leaf content of Na, Ca, and Cl was investigated. As seen in Table 4, the Ca content was affected relative to the Ca concentration in the nutrient solution (NS). In the leaf tissues, the highest concentration was found in both the HCa and LCa treatments, followed by the rest. The Na content was also relevant to the concentration of the NS, with the HNa treatment to accumulate the most, followed by LNa, while the rest of the treatments were unaffected compared to the control. Finally, the Cl content of the leaves appeared to be significantly affected and relevant to the amount of chloride in the NS. Hence, the highest concentration was observed in the HCa, followed by the HNa. In addition, the Cl content of the HNa treatment did not differ from either of the Ca treatments, whereas LNa was significantly lower than HNa, and greater compared to the control, which had the lowest values compared to all the rest.

**Figure 4:**
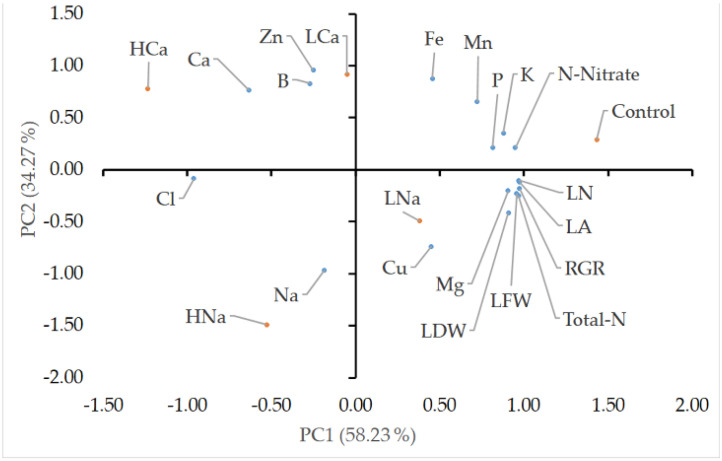
Principal component loading plot (PCA) of agronomical characteristics (LFW: leaf fresh weight, LDW: leaf dry weight, LN: leaf number, LA: leaf area, RGR: relative growth rate) and nutrient content (N-Nitrate: Nitrate, Total-N: Leaf Total Kjeldahl nitrogen, Mg: magnesium, K: potassium, P: phosphorus, Cu: Copper, Zn: Zinc, Fe: iron, Mn: manganese, B: boron, Ca: calcium, Na: sodium, and Cl: chloride content in dry leaf tissues) of corn salad plant (*Valerianella locusta*) grown in a floating system at harvest stage, as influenced by four salinity treatments (referred to as low and high salinity level) and the salinity source. Control: standard nutrient solution (−0.08 MPa, 20 °C); LNa: low NaCl-salinity (−0.18 MPa, 20 °C); HNa: high NaCl-salinity (−0.28 MPa, 20 °C); LCa: low CaCl_2_-salinity (−0.018 MPa, 20 °C); HCa: high CaCl_2_-salinity (−0.28 MPa, 20 °C). The mean values were used in each situation. Percentage values of the principal components (PC1 and PC2) indicate variation explained by each PC.

In the principal component (PC) analysis carried out for the agronomical characteristics and nutrient content of corn salad, cultivated in a control NS and four salinity treatments differing in their osmotic potential level and salinity source, two highly independent groups were observed, indicating that the first two PCs clarified 92.5% of the total variance, with PC1 and PC2 resulting in 58.23% and 34.27%, respectively (Figure 4). PC1 was positively correlated with all the agronomical characteristics (LFW, LDW, LA, LN, and RGR) and all the nutrients except for Ca, Na, Cl, Zn, and B. On the other hand, PC2 was positively correlated with Ca, B, Zn, Fe, Mn, P, K, and NO_3_ content, whereas Total-N, Cl, Na, Cu, Mg, and all the agronomical characteristics were negatively correlated with it. According to the graphical representation, corn salad cultivated in CaCl_2_ eustress condition, regardless of the EC, is placed in the upper left quadrant, even though the high EC treatment (HCa) is further to the left compared to the lower EC (LCa). On the contrary, the NaCl eustress treatments are not placed in the same quadrant, with the high EC treatment (HNa) placed in the lower left quadrant and the lower EC treatment (LNa) placed int the lower right quadrant. Finally, the control treatment was placed in the upper right quadrant.

## 3. Discussion

Corn salad (*Valerianella locusta* L.), is a leafy vegetable that is gaining much interest as an ingredient in fresh-cut salads [6,9,10,71]. On the other hand, leafy vegetables are often accused of having a high nitrate content that can result from a higher nitrate uptake rate than the assimilative capacity of the plant, which can be dangerous for human health [72]. Among the alternative horticultural and agronomic practices that can limit the nitrate accumulation without compromising the crops’ performance is the use of salinity as a “eustressor” [73,74]. Given that most horticultural crops are non-halophytes and lack the genetic background that would allow them to adapt, tolerate, or benefit from salt stress through their physiological mechanisms [75], the “salinity-eustress” has to be applied in a controlled way, with a certain type of salt, dosage, and sometimes even duration, so that the cultivation’s quality will benefit without compromising the yield [76,77,78]. In this study, the effects of the addition of different salt solutions (NaCl and CaCl_2_) that were isosmotic on two levels (high and low), on agronomical performance as well as nitrate content and mineral composition, of hydroponically grown corn salad were explored.

### 3.1. Growth and Productivity Response

To assess the level of yield reduction, the agronomical characteristics of corn salad were measured at the harvest stage, namely, the leaf fresh and dry weight (LFW and LDW), leaf number (LN), and leaf area (LA). As seen in Figure 1a,b, corn salad proved to be a rather susceptible plant species to long-term osmotic stress applied after the transplanting stage. The type of salt also played an important role, since the stress that was induced by the bivalent inorganic salt, CaCl_2_, appeared to be more severe, regardless of the EC level, suggesting the involvement of chloride in the yield reduction. On the other hand, the monovalent inorganic salt, NaCl, did not impair any of the agronomical parameters under low EC conditions. The importance of the osmotic potential on fresh and dry weight has been demonstrated by several studies, most of which focus on the osmotic stress induced by the presence of NaCl, probably due to its association with low irrigation water quality [52,79,80,81,82,83,84,85], and few explore the effects of different salt sources such as KCl, Na_2_SO_4_, or CaCl_2_ [86,87,88,89]. From these studies, the results of Ntatsi et al. [86], who experimented on a salinity tolerant plant, *Cichorium spinosum*, also showed that under high salinity levels, CaCl_2_ had a stronger effect on dry weight, even though under low salinity all the salts decreased both fresh and dry weight equally. On the other hand, Corrado et al. [87], when studying different lettuce cultivars under isosmotic solutions of NaCl- and CaCl_2_-induced eustress, did not observe an influence of the type of salt on yield reduction, and rather considered the osmotic level as the responsible factor. Carillo et al. [88] expected that the application of CaCl_2_ would surpass both the osmotic and ion-specific effects of NaCl, reducing “baby” lettuce yield, but this was not observed in their study, perhaps due to the short-term production of “baby leaves”. Apart from reduced fresh and dry weight, a reduction of the LN and LA was also observed. As seen in Figure 1c,d, LN and LA followed the exact same differences, with the highest values observed in the control, followed by the LNa, which was also similar to HNa, which in turn did not differ significantly from the LCa. As observed for other parameters, HCa was again the treatment with the lowest values. On the one hand, a reduction in LA was expected since leaf expansion is related to cell elongation, which is affected by cellular water uptake and cell osmotic potential, both of which are affected by the higher osmotic potential of a growth medium [90]. On the other hand, the LN was also reduced in relation to the osmotic potential and type of salt, suggesting that the leaf initiation rate was also affected and that, in the given context, cell division and tissue expansion functioned as a coupled process [91].

Moreover, in Figure 2, it is clear that the RGR was also affected by the treatments applied. The control had the highest values, followed by LNa, which in turn also had similar values to HNa and LCa. HCa was the treatment where the lowest values were calculated. In addition, several researchers who studied the cause of the growth reduction in plants grown under high external NaCl conditions [92,93,94] observed that the photosynthesis per leaf area was mostly unaffected, and therefore not per se responsible for the growth reduction of the plants. Therefore, even though it can often be challenging to discriminate the effects of osmotic stress and ion toxicity and their share on the yield reduction of different horticultural crops or even cultivars [86], we suggest that both the osmotic potential and chloride concentration of the NS affected the growth of corn salad through the reduction of leaf expansion, due to reduced LA and LN, which in turn resulted in a lower photosynthesis per plant basis, as also observed by Munns and Tester [95].

### 3.2. Nutritional Content of Leaf Tissues

In an effort to understand the long-term effect of an applied type of eustress on growth (e.g., its osmotic potential and salt source), the nutrient content of leaves of corn salad was analyzed. Due to the complexity of nutrient interactions, cultivar-related characteristics, harvest stage, plant density, and parameters related to the growing method, the outcome of the plant tissue analysis is not yet completely understood. Several studies focus on the production of baby-leaf *Valerianella locusta*, which presupposes a plant density of 880 plants m^−2^ or more [27,96,97,98]; since the plant density used in this study was 53 plants m^−2^ and the harvest stage was different (approximately 15 pairs of leaves of the control instead of 3–8), it is expected that the nutrient content might differ from that of other studies.

#### 3.2.1. Nitrates and Total Kjeldahl Nitrogen

Regarding the nitrate levels (Table 2), the highest content was observed in the control, followed by LCa and LNa, with the latter having similar values to HNa, which however had significantly less nitrate compared to the LCa. Moreover, the nitrate content of HNa was similar to that of HCa, which was the treatment with the lowest values compared to all the other treatments. The physical properties of chloride are similar to nitrate, deeming their relation antagonistic since the nitrate transporter Z*m*NPF6.4, a transmembrane protein located in the root, is not able to discriminate between the two [99]. Since the reduction of nitrates in the leaf tissues was observed in the treatments with higher chloride concentration in the nutrient solution, our results could be associated with the antagonism of nitrate and chloride, rather than the osmotic potential of the nutrient solution itself, and are in agreement with the findings of other researchers [100,101,102,103,104].

Reducing the nitrate content of leafy vegetables is considered important for human health after the raising of several concerns in recent decades [30,31,32]. These concerns are hard to prove either way, due to inconsistent results of epidemiological studies that explore the association of dietary nitrate and different types of cancer [105,106,107,108,109]. Hence, in a “better safe than sorry” approach, certain thresholds for different leafy vegetables have been set by the European Commission, as stated in the *Official Journal of the European Union*, Commission Regulation (EU) No 1258/2011 [110]. These thresholds refer to spinach, different types of lettuce, and rucola, and are set from approximately 2000 to a maximum of 5000 mg of NO_3_ per kg of fresh weight, depending on the vegetable and season of reference. In the scope of protecting human health and keeping up with EU regulations, it has been suggested from several studies that the demonstrated antagonistic relations of nitrate and chloride can lead to a decreased nitrate content in leafy vegetables and, in several cases, this could be utilized for the production of “safer” foods [73,77,81,88]. Corn salad is considered a species with a high nitrate content (>2500 mg kg^−1^ of fresh weight) but its thresholds are not yet defined in the EU regulations [13,27,28]. As a result, even though it has been found that maintaining nitrate levels under 2000 mg kg^−1^ of fresh weight is possible for corn salad, different EU countries apply different maximum limits, that are either mandatory or advisable [111,112]. Therefore, it is also important to note that the nitrate content of all the treatments explored in this research, when transferred to LFW (data not sown), were always below the 2000 mg kg^−1^ of fresh weight threshold. Hence, in combination with the yield results, corn salad could be grown hydroponically, as described in this study under mild NaCl eustress conditions (NaCl 20 mM), with both safe nitrate levels and high yield. 

Regarding the total Kjeldahl N levels in Table 2, no significant differences were observed between the four salt solutions and the control. It has been previously demonstrated by other researchers that salinity induced by NaCl can reduce the nitrate content without affecting the total N content, though the reason behind this observation is not completely understood [113,114,115]. More recently, similar to our results, Bres et al. [116] found that in hydroponically grown lettuce, the nitrate content was reduced with the addition of 20 and 40 mM of NaCl in the NS, but total N was not affected.

#### 3.2.2. Non-Treatment-Related Macronutrients (Mg, K, P) and Micronutrients (Cu, Zn, Fe, Mn, B)

The four saline solutions were prepared through the addition of different mM of NaCl and CaCl_2_ to the control solution. Hence, any observed differences in the concentration of macronutrients such as Mg, K, and P in the leaf tissues would be ascribed to the presence of additional salts and their dosage in the NSs. Nevertheless, no significant differences were observed for the Mg, K, and P content of the plant leaf tissues (Table 3), suggesting that the additional Na, Ca, and Cl in the nutrient solution did not interfere with the uptake of these three macronutrients. Towards that end, it was expected that Mg uptake would be depressed by the increased Ca concentration of the CaCl_2_ treatments, or perhaps affected by the Na and K antagonistic relations [117,118]. Nevertheless, the additional Ca did not interfere with Mg uptake. In the same direction, it has been demonstrated by researchers that under NaCl salinity stress, Na and K ions compete for binding on the plasma membrane due to their chemical similarity, which results in reduced K influx and decreased K content in the leaf tissues [119,120,121,122]. We speculate that the abundance of K ions in the NS overshadowed the effect of its antagonistic relations with Na, as it perhaps also happened with Mg and Ca. Ciriello et al. [81] observed that in basil plants that were subjected to different NSs with NaCl or NaCl + CaCl_2_, Mg content increased under high NaCl stress, whereas K concentration remained unaffected and P content decreased under high ECs regardless of the salt mix. In contrast with these results, Mg concentration was reduced in *Cichorium spinosum* L. grown under increased NaCl conditions, while K was unaffected and P content increased [84]. In further research on *C. spinosum* L. [86], the Mg content again reduced under NaCl rather than CaCl_2_ conditions, though in contrast with previous results, the leaf K content was unaffected under 40 mM NaCl and 26.7 mM CaCl_2_, whereas P was unaffected this time. In addition, results from Carillo et al. [88] regarding the cultivation of lettuce in a floating system showed that the salinity source altered the nutrient content of Mg where the control and NaCl conditions maintained high concentrations, compared to K, which increased under KCl conditions relative to the NS concentration, and P which in turn was significantly reduced in response to CaCl_2._ The values of the macronutrients presented in this study are far greater compared to a study by Gottardi et al. [97] and another by Radaan et al. [98], but that is ascribed mainly to the plant density in those experiments, which was 1800 and 2200 plants m^−2^, respectively, and, secondly, to differences in the nutrient solution.

Table 4 shows that only the boron content measured in dry leaf tissues was affected between the treatments. Towards that end, B content was greater in the leaf tissues of LCa and lowest in the HNa, whereas the B content of the control, LNa, and HCa did not differ from the aforementioned treatments. This observation could perhaps be partially explained through the relations of Na, Ca, and B on cell expansion and pectin [123,124], as described in the next section. Since the interactions and relations between the elements are complex, especially when it comes to micronutrient interactions [125,126,127,128], more research should be conducted before drawing conclusions in relation to the osmotic potential, nutrition, and Na, Cl, and Ca on hydroponically cultivated corn salad.

#### 3.2.3. Treatment-Related Nutrients and Non-Nutrients (Ca, Na and Cl)

Since the differences among the treatments were related to the concentration of Na, Ca, and Cl in the NSs through the addition of NaCl or CaCl_2_ until two EC levels were reached, while maintaining isosmotic conditions for the differences on each level (see Table 5), the uptake of these elements was where the key differences were expected to be observed. As expected, Ca, Na, and Cl leaf content increased in relation to their abundance in the nutrient solution, though Cl content was observably higher in the leaf tissues of the CaCl_2_ treatments compared to their isosmotic NaCl EC levels. These results suggest that the yield reduction might indeed be attributed to the Cl content rather that Na, Ca, or the osmotic potential. It is often suggested by other researchers that the LFW reduction derives from the negative effects of Na on the photosynthetic rate of plants [77,84,129]. Moreover, given that the Na toxicity is dependent on the K/Na ratio in the cell, and its capacity to compartmentalize this ion in the vacuole and not on its absolute amount in the cytosol, the unaffected K concentration of the leaf tissues of the LNa and HNa treatments could have played a role in alleviating the NaCl salinity stress [130]. Given that Ca is an important nutrient for plant growth and signal under various stress conditions [131,132,133,134,135,136], it is not expected to be treated as a stress factor by the plants and, as a result, it is not considered to be responsible for the acute reduction of the LFW of the CaCl_2_ treatments. Nevertheless, the greater yield reduction was observed in the LCa and HCa treatments. Under NaCl conditions, Ciriello et al. [81] demonstrated the beneficial effects of additional Ca towards the alleviation of salinity stress. Even though the interactive effects of Na and Ca on cell wall extensibility, synthesis, plasmalemma function, and cell turgor are not clear, the partial reverse of the deleterious effects of NaCl-induced salinity on root growth by supplemental Ca on cotton seedlings is attributed to cell elongation at the expense of radial expansion, and maintained rates of cell production [137]. When Ca is applied exogenously, it can also regulate K/Na selectivity, conferring salt adaptation by improving signal transduction [138]. On the other hand, increased Na in the nutrient solution can also trigger a calcium-dependent signaling pathway that starts from Ca sensor protein calcineurin B-like 4 (CBL4) and leads to Na efflux from the cytosol [139]. Moreover, a link between plant–surface lipids and Ca influx has been suggested as a result of salt sensing in plants [140]. Towards that end, there is a direct and indirect relationship between Na, Ca, B, and cell expansion through the rhamnogalacturonan II component of pectin [123,124,141]. Nevertheless, the growth of the NaCl treatments were not as affected as the CaCl_2_ treatment, indicating that the negative effect of growth cannot be ascribed to Na or Ca.

Hence, our observations suggest that the negative effect on plant growth should be ascribed to the Cl accumulation rather than the presence of Na or Ca in the nutrient solution. In fact, it has generally been accepted that Cl concentration levels between 4 and 7 mg g^−1^ of dry leaf tissues are toxic for Cl-sensitive plants [142]. Early research by Cramer and Spurr et al. [143] suggested that Cl had a positive effect on alleviating Na stress on hydroponically grown lettuce when comparing isosmotic solutions with added Na_2_SO_4_ and NaCl. More recent findings indicate that the growth inhibition observed in the presence of Na_2_SO_4_ is attributed to SO_4_^2−^ and not Na [144]. Therefore, it is safe to discard the notion of the stress-alleviation effect of Cl in our case. In fact, several studies have demonstrated the opposite. Mild and moderate CaCl_2_ concentrations can lead to more intense phytotoxic effects than NaCl due to the bivalent nature of the former, the increased chloride concentration, and its toxic effects [86,145,146]. Moreover, Cl uptake and transport from the roots to the leaves is not as controlled as that of Na, thus drastically affecting plant metabolism and development [147,148]. Nevertheless, the effect of Na, Cl, and Ca on the morphology, photosynthetic capacity, and ion accumulation might differ between plant species and can be a result of different salinity acclimation strategy [145,149,150,151].

## 4. Materials and Methods

### 4.1. Plant Material and Cultivation Conditions

This work was conducted on *Valerianella locusta* L. “Elixir” (HM.CLAUSE Sas, Portes-Les-Valence, France). The experiment was carried out during the 2021 autumn growing season (from 15 September to 5 November 2021) inside a heated greenhouse of the Laboratory of Vegetable Crops at the Agricultural University of Athens, located in central Greece (37°58′57.8″ N, 23°42′14.3″ E). On 15 September 2021, “Elixir” was sown on rockwool sheets (200 cubes per sheet, AO Plug, Grodan, Roermond, the Netherlands), covered with a thin layer of vermiculite, and placed on stainless steel benches to germinate. During the germination period, the rockwool sheets were placed in the main chamber of the greenhouse, with ambient light conditions, and temperatures between 15 °C and 27 °C during the day and 10 °C and 19 °C during the night. After 22 days, the produced seedlings were placed into plastic net pots and transplanted in the 15 floating rafts. The rafts were previously cut from polystyrene trays (EPS 30, 12 kg/m^3^) and each board was 3 cm thick. The plant density was 53 plants per m^2^ and each hole had a 5 cm diameter. The holes were made manually and the cut material was thrown away. Prior to the crop establishment, the rafts were cleaned with diluted CaCl_2_ solution, rinsed, and left to dry in the sun before being used for this experiment. The 15 floating tanks (FT) used in this experiment were constructed from stainless steel (IntelAgro, Thermi, Greece) and were 30 cm deep, 55 cm wide, and 180 cm long. Each FT had a constant volume of 180–200 L of fresh nutrient solution (NS), which was adjusted by a floater device connected to a 50 L replenishment tank (RT) which maintained a stable water level by dripping the needed amount of the same NS (Figure S1). For this reason, the area of the tank where the floater was connected did not accommodate any plants, and the surface was covered with Styrofoam to avoid algae growth due to sunlight reaching the NS. In each experimental unit, the dissolved oxygen (O_2_) level was maintained above the 6 mg L^−1^ threshold limit through the combination of an air stone and an immersion pump that recirculated the nutrient solution inside each FT. All experimental units were arranged in a randomized complete block design with three replicates per treatment. Each experimental unit was an FT which accommodated a total of 48 plants. A comparison of five nutrient solutions—a non-salinized control and four saline solutions—is described in the next section. 

### 4.2. Nutrient Solution Composition and Iso-Osmotic Salt Application per EC Level

NUTRISENSE (accessed on 1 September 2021, https://nutrisense.online/), an online Decision Support System (DSS) program, was used for the determination of the composition of each nutrient solution treatment and the quantities of the fertilizers required [152] (Laboratory of Vegetable Production, Agricultural University of Athens, Athens, Greece). The final solution was prepared by diluting the dense A and B nutrient solutions 100 times and adding nitric acid to reach the chosen EC levels and pH values. The dilution took place in a 300 L barrel that was connected with a pump that either recirculated the solution or pumped it via rubber tubing into the experiment chamber’s tanks. The chemical composition of the basic NS (control) was K: 6.00 mmol L^−1^, Ca: 5.50 μmol L^−1^, Mg: 2.00 μmol L^−1^, NO_3_^−^: 14.00 μmol L^−1^, NH_4_^+^: 0.92 μmol L^−1^, SO_4_^2−^: 3.11 μmol L^−1^, H_2_PO_4_^−^:1.50 μmol L^−1^, Fe: 25.00 μmol L^−1^, Mn: 10.00 μmol L^−1^, Zn: 7.00 μmol L^−1^, Cu: 0.80 μmol L^−1^, B: 35.00 μmol L^−1^, and Mo: 0.60 μmol L^−1^, and it was monitored every two days. The electrical conductivity (EC), osmotic potential, and pH of the control NS were 2.47 dS m^−1^, −0.08 MPa at 20 °C, and 5.6, respectively. The other NSs were prepared by adding different amounts of sodium chloride (NaCl) and calcium chloride (CaCl_2_) to the basic NS, so that the final solutions were isosmotic on each EC level. The osmotic potential (Ψs) at 20 °C was −0.18 MPa and −0.28 MPa in the low- and high-salinity level, respectively, and was calculated by using the Van ’t Hoff equation Ψs = −cRT, where c is the solute concentration in mol L^−1^, R is a constant (8.3 × 10^−3^ L MPa mol^−1^ K^−1^), and T is temperature in Kelvins (°K) [153]. By adding 20 mM and 40 mM of NaCl to the basic NS, the low and high EC sodium chloride treatments were achieved (LNa and HNa). In like manner, by adding 13.3 mM and 26.5 mM of CaCl_2_ to the basic NS, the low and high EC calcium chloride treatments were achieved (LCa and HCa). This addition of salts led to two total ionic concentrations and four different EC levels, as shown in Table 5 and Table 6. The NS in all treatments was replaced weekly to guarantee the same initial mineral nutrient conditions (Table S1).

### 4.3. Sampling, Growth, Yield, and Leaf Biomass Determination

When the control treatment reached what was considered the commercial stage (29 days after transplanting, 5 November), all the plants per FT were harvested to determine the leaf fresh weigh of the crop (LFW; expressed in g per plant) by measuring each plant on a Mettler PE 3600 balancer (Mettler-Toledo, Columbus, OH, USA). Apart from the LFW, five plants from each tank were chosen to also be used for the measurement of the total leaf area (LA; expressed in cm^2^ per plant), which was conducted by separating the leaves by hand and placing them on the transparent belt of LI-3100C (LI-COR, Inc. Lincoln, NE, USA) and the leaf number (LN; expressed as leaf number per plant) which was determined by simultaneously counting the leaves placed on the belt. The separated leaves, along with the stem, were then stored in a paper bag and placed in a drying oven (STF-N 400, FALC Instruments S.L.R, Treviglio, Italy) at 65 °C for 3 days in order to dry the leaf tissues until they reached a constant weight and measure the corresponding dry weight (LDW; expressed in g per plant). At the beginning of the experiment, 50 seedlings were also sampled to estimate the fresh and dry weight of the plants before being transplanted to the FTs. With those data, the relative growth rate (RGR; expressed as g day^−1^) was calculated using the equation reported by De Groot et al. [154]:

RGR = (lnW2 − lnW1)/(t2 − t1)(1)

where W1 and W2 are the LFWs (g) of the shoots at given dates t1 and t2 (days), corresponding to the beginning and the end of the experiment, respectively.

For the determination of the mineral composition, 10 of the harvested plants per FT (constituting one replicate) were weighted and stored in paper bags and dried to ensure that the necessary grams of dry plant tissue would be available for the following chemical analysis. The dried corn salad material was then milled and sieved at the highest speed option (6000–6500 rpm) by passing it through the MF 10 Microfine grinder (IKA Werke, Staufen, Germany) and collecting the grated tissues in sealable plastic bags.

### 4.4. Essential Nutrients, Sodium, and Chloride Determination

Dried and ground leaf tissues were processed with the dry ashing method, for the determination of the nutrient content. In particular, 0.5 g of dry plant were turned into ash by adding them in porcelain cups and then placing them in chamber furnace LM-112 (Linn High Therm, Hirschbach, Germany) for 8 h at 500 °C. After this step, 10 mL of HCl solution (0.25 N) were added in the porcelain cups. The cup content was then filtered through 125 mm Macherey-Nagel filter papers and transferred in 100 mL volumetric flasks. To produce the aqueous tissue extracts (ATE), the flasks were filled with distilled water until the solution level reached 100 mL. The ATEs were then transfused in 100 mL plastic bottles and stored in a refrigerator until the chemical analysis was carried out.

The nutrient concentration of the ATEs was conducted with various methods depending on the element. The colorimetric molybdenum blue reaction was used for the determination of phosphorus (P) and the photometry was carried out at 880 nm [155,156] in the Anthos Zenyth 200 (Biochrom Ltd., Cambridge, UK). For the quantification of calcium (Ca), magnesium (Mg), iron (Fe), manganese (Mn), and zinc (Zn), the measurements were conducted through Flams Atomic Absorption Spectroscopy (FASS) [157] using the Atomic Absorption Spectrometer Shimadzu AA-7000 (Shimadzu, Kyoto, Japan). The calibration curve was obtained by using standard solutions of the respective metallic salts (1000 ppm). For the operation of AA-700, the acetylene gas flow was set to 1.5 L/min and vacuum pressure to 3.5 bar. Sodium (Na) and Potassium (K) were measured through flame photometry [158] by placing the ATEs in the Sherwood Flame Photometer 410 (Sherwood, Cambridge, UK). Boron (Β) content was determined spectrophotometrically with an azomethine H derivative and photometering at 420 nm using the Anthos Zenyth 200 [159,160]. For the determination of total Nitrogen (Τotal-Ν), the Kjeldahl method was selected. The digestion and distillation were carried out on Labtec DT 220, simultaneously used with Scrubber Labtec SR 210, and Tecator Kjeltec 8200 ([161], FOSS A/S, Hillerød, Denmark), respectively. After manually titrating each distilled sample by measuring the volume (ml) of HCL solution (0.05 N) needed to turn the solution’s color from green to pink, the Τotal-N determination was completed. The nitrate content was determined colorimetrically from dry leaf tissues [162], by nitration of salicylic acid and photometering at 410 nm using the Anthos Zenyth 200. For the determination of chloride, the Iwasaki assay was used [163]. The quantity of elements present in the ATEs was expressed as the means of the elemental concentration of three replicates.

### 4.5. Statistical Analysis

All experimental data were subjected to One-Way ANOVA using the Statistica 12 software package for windows (StatSoft Inc., Tulsa, OK, USA). Duncan’s multiple range test was performed at a *p* ≤ 0.05 level on each of the significant variables measured. Data are presented in graphs as the means ± SE of three replicates. Principal component analysis (PCA) was performed on the entire dataset with IBM SPSS statistic software v. 26.0 (Armonk, NY, USA).

## 5. Conclusions

Different eustress conditions were applied throughout the hydroponic cultivation of corn salad (*Valerianella locusta* “Elixir”) to evaluate the effect of two different types of salts and their dosage in the scope of determining a eustress solution that could be utilized without reducing the productivity of the crop. Sodium chloride, NaCl, had a less severe effect on the agronomical characteristics of corn salad compared to calcium chloride, CaCl_2_, when compared at isosmotic levels, suggesting that the type of salt had a significant effect over the osmotic potential. Nitrate concentration was also reduced more under a CaCl_2_ eustress, which was attributed to the antagonistic relations of nitrate and chloride. Total Kjeldahl Nitrogen was unaffected, as were most of the macro and micronutrients. On the other hand, leaf sodium, calcium, and chloride content appeared to increase in relation to their concentration in the treatment’s NSs. In conclusion, low-quality water, containing up to 20 mM NaCl, could be utilized for the hydroponic cultivation of corn salad and when eustress is considered, NaCl should be the salt of choice rather than CaCl_2_.

## Figures and Tables

**Table 1:** Nitrate (NO_3_) content and Total-N content (mg g^−1^ of dry weight (LDW)) in the leaves of *Valerianella locusta* plants grown in a floating system, as influenced by four salinity treatments (referred to as low and high salinity level) and the salinity source. Control: standard nutrient solution (−0.08 MPa, 20 °C); LNa: low NaCl-salinity (−0.18 MPa, 20 °C); HNa: high NaCl-salinity (−0.28 MPa, 20 °C); LCa: low CaCl_2_-salinity (−0.018 MPa, 20 °C); HCa: high CaCl_2_-salinity (−0.28 MPa, 20 °C).

Treatment	Leaf NO_3_ (mg g^−1^ LDW)	Leaf Total-N (mg g^−1^ LDW)
Control	13.85 ± 0.338 a	55.70 ± 1.5
LNa	10.66 ± 0.782 bc	54.91 ± 1.3
HNa	9.72 ± 0.202 bc	57.52 ± 0.8
LCa	11.15 ± 0.726 b	54.40 ± 1.1
HCa	8.981 ± 0.521 c	53.44 ± 0.6
Statistical Significance	*	NS

Values represent means ± standard error of three replicates. Means within the same column followed by different letters indicate significant differences according to Duncan’s multiple range test: * indicates significance at *p* < 0.05; NS = not significant.

**Table 2:** Mg, K, and P (mg g^−1^ of leaf dry weight (LDW)) in the leaves of *Valerianella locusta* plants grown in a floating system, as influenced by four salinity treatments (referred to as low and high salinity level) and the salinity source. Control: standard nutrient solution (−0.08 MPa, 20 °C); LNa: low NaCl-salinity (−0.18 MPa, 20 °C); HNa: high NaCl-salinity (−0.28 MPa, 20 °C); LCa: low CaCl_2_-salinity (−0.018 MPa, 20 °C); HCa: high CaCl_2_-salinity (−0.28 MPa, 20 °C).

Treatment	Mg (mg g^−1^ LDW)	K (mg g^−1^ LDW)	P (mg g^−1^ LDW)
Control	3.46 ± 0.22	59.50 ± 1.5	10.30 ± 0.46
LNa	3.26 ± 0.26	57.33 ± 4.05	8.68 ± 0.91
HNa	3.25 ± 0.30	51.33 ± 2.67	8.93 ± 0.56
LCa	3.18 ± 0.29	58.00 ± 2.00	9.30 ± 0.12
HCa	3.13 ± 0.13	51.33 ± 1.33	8.60 ± 0.53
Statistical Significance	NS	NS	NS

Values represent means ± standard error of three replicates. NS indicates no significant differences according to Duncan’s multiple range test (*p* < 0.05).

**Table 3:** Cu, Zn, Fe, Mn, and B (μg g^−1^ of dry weight (LDW) in the leaves of *Valerianella locusta* plants grown in a floating system, as influenced by four salinity treatments (referred to as low and high salinity level) and the salinity source. Control: standard nutrient solution (−0.08 MPa, 20 °C); LNa: low NaCl-salinity (−0.18 MPa, 20 °C); HNa: high NaCl-salinity (−0.28 MPa, 20 °C); LCa: low CaCl_2_-salinity (−0.018 MPa, 20 °C); HCa: high CaCl_2_-salinity (−0.28 MPa, 20 °C).

Treatment	Cu (μg g^−1^ LDW)	Zn (μg g^−1^ DW)	Fe (μg g^−1^ DW)	Mn (μg g^−1^ DW)	B (μg g^−1^ DW)
Control	12.40 ± 097	119.37 ± 6.24	73.81 ± 10.23	202.61 ± 49.44	1.22 ± 0.218 ab
LNa	13.93 ± 1.73	116.98 ± 9.24	58.20 ± 9.95	181.49 ± 33.17	1.43 ± 0.104 ab
HNa	13.16 ± 1.28	113.09 ± 12.28	44.80 ± 1.29	130.39 ± 10.12	1.03 ± 0.149 b
LCa	12.04 ± 0.88	126.87 ± 1.35	68.47 ± 13.60	193.67 ± 41.32	1.84 ± 0.198 a
HCa	10.88 ± 1.33	125.76 ± 10.27	65.09 ± 1.31	159.31 ± 1.99	1.62 ± 0.215 ab
Statistical Significance	NS	NS	NS	NS	*

Values represent means ± standard error of three replicates. Means within the same column followed by different letters indicate significant differences according to Duncan’s multiple range test (*p* < 0.05): * indicates significance at *p* < 0.05; NS = not significant.

**Table 4:** Na and Ca content (mg g^−1^ of dry weight (LDW)) in the leaves and Cl (mg g^−1^ of dry weight (LDW)) in the leaves of *Valerianella locusta* plants grown in a floating system, as influenced by four salinity treatments (referred to as low and high salinity level) and the salinity source. Control: standard nutrient solution (−0.08 MPa, 20 °C); LNa: low NaCl-salinity (−0.18 MPa, 20 °C); HNa: high NaCl-salinity (−0.28 MPa, 20 °C); LCa: low CaCl_2_-salinity (−0.018 MPa, 20 °C); HCa: high CaCl_2_-salinity (−0.28 MPa, 20 °C).

Treatment	Ca (mg g^−1^ LDW)	Na (mg g^−1^ LDW)	Cl (mg g^−1^ LDW)
Control	3.51 ± 0.78 b	0.66 ± 0.10 c	1.86 ± 0.17 d
LNa	4.15 ± 1.20 b	2.50 ± 0.32 b	6.28 ± 0.5 c
HNa	3.58 ± 0.46 b	4.96 ± 0.57 a	8.32 ± 0.57 ab
LCa	13.69 ± 0.76 a	0.74 ± 0.11 c	7.48 ± 0.32 b
HCa	15.57 ± 3.69 a	0.52 ± 0.05 c	9.13 ± 0.42 a
Statistical Significance	*	*	*

Values represent means ± standard error of three replicates. Means within the same column followed by different letters indicate significant differences according to Duncan’s multiple range test (*p* < 0.05): * indicates significance at *p* < 0.05.

**Table 5:** Addition of different salts (mM) to a basic nutrient solution (control) aiming to establishing four salinity treatments (referred to as low and high salinity level) and the salinity source. Control: standard nutrient solution (−0.08 MPa, 20 °C); LNa: low NaCl-salinity (−0.18 MPa, 20 °C); HNa: high NaCl-salinity (−0.28 MPa, 20 °C); LCa: low CaCl_2_-salinity (−0.018 Mpa, 20 °C); HCa: high CaCl_2_-salinity (−0.28 MPa, 20 °C).

Treatments	NaCl (mM)	CaCl_2_ (mM)	Total Ionic Conc. (mM)	EC dS/m	Ψs (MPa at 20 °C)
Control	0	0	34.51	2.47	−0.08
LNa	20	0	74.51	4.74	−0.18
HNa	40	0	114.51	7.04	−0.28
LCa	0	13.3	74.49	5.22	−0.18
HCa	0	26.5	115.51	7.94	−0.28

**Table 6:** Chemical composition of the 5 treatments. All 5 solutions were based on the same control solution and the differences in their chemical composition are caused only by the addition of Ca, Na, and Cl in the form of NaCl and CaCl_2_.

Element Concentration (mM)	Control	LNa	HNa	LCa	HCa
K	6.00	6.00	6.00	6.00	6.00
Ca	5.50	5.50	5.50	18.83	32.17
Mg	2.00	2.00	2.00	2.00	2.00
NH_4_	0.92	0.92	0.92	0.92	0.92
SO_4_	3.11	3.11	3.11	3.11	3.11
NO_3_	14.00	14.00	14.00	14.00	14.00
H_2_PO_4_	1.50	1.50	1.50	1.50	1.50
Fe	0.0250	0.0250	0.0250	0.0250	0.0250
Mn	0.01	0.01	0.01	0.01	0.01
Zn	0.007	0.007	0.007	0.007	0.007
Cu	0.0008	0.0008	0.0008	0.0008	0.0008
B	0.04	0.04	0.04	0.04	0.04
Mo	0.0006	0.0006	0.0006	0.0006	0.0006
Cl	0.40	0.40	0.40	27.05	54.73
Na	0.60	0.60	0.60	0.60	0.60
HCO_3_	0.40	0.40	0.40	0.40	0.40
NaCl		40.00	80.00		

## Data Availability

Not applicable.

## Supplementary Materials

The following supporting information can be downloaded at: https://www.mdpi.com/article/10.3390/plants12071454/s1, Figure S1: View of the cultivation of corn salad in floating raft systems during the final week of the experiment. Table S1: The chemical composition of the nutrient solutions was measured over time (days from transplanting, DFT) prior to their renewal. The same solution as the one in DFT 0 was used for each renewal. On DFT 7, samples were taken and the solutions were renewed. The next emptying and refilling occurred at DFT 18 and DFT 28 during the harvest stage.
