# Supplementary material for: Effects of NaCl and CaCl2 as Eustress Factors on Growth, Yield, and Mineral Composition of Hydroponically Grown Valerianella locusta
Source: Plants (Basel). 2023 Mar 26;12(7):1454. doi: 10.3390/plants12071454 (PMC10097257; doi:10.3390/plants12071454)
Supplement: Supplementary file 1 [file plants-12-01454-s001.zip › Table S1.pdf]

Table S1. The chemical composition of the nutrient solutions was measured over time (days from transplanting, DFT) prior to their renewal. The same solution, as the one in DFT 0, was used for each renewal. On DFT 7, samples were taken and the solutions were renewed. The next emptying and refilling occurred at DFT 18 and DFT 28 during the harvest stage.

	DFT	DFT 0	DFT 7	DFT 18	DFT 28	Statistical analysis
Control	K (mmo/l)	6.00	6.45	6.70	6.50	ns
	Ca (mmol/l)	5.50	5.45	5.40	5.70	ns
	NO_3 (mmol/l)	14.00	15.12	13.85	14.38	ns
	P (mmol/l)	1.50	1.4945	1.49525	1.571	ns
	Na (mmol/l)	0.60	0.75	0.59	0.61	ns
LNa	K (mmo/l)	6.00	6.70	6.67	6.58	ns
	Ca (mmol/l)	5.50	5.55	5.64	6.89	ns
	NO_3 (mmol/l)	14.00	15.00	13.90	13.70	ns
	P (mmol/l)	1.50	1.51	1.49	1.49	ns
	Na (mmol/l)	20.00	19.71	20.34	21.10	ns
LCa	K (mmo/l)	6.00	6.28	6.40	6.00	ns
	Ca (mmol/l)	18.83	19.00	18.98	18.40	ns
	NO_3 (mmol/l)	14.00	15.29	13.78	13.20	ns
	P (mmol/l)	1.50	1.56	1.51	1.53	ns
	Na (mmol/l)	0.60	0.70	0.89	0.85	ns
HNa	K (mmo/l)	6.00	6.15	6.36	6.20	ns
	Ca (mmol/l)	5.50	5.54	5.59	5.34	ns
	NO_3 (mmol/l)	14.00	15.20	13.57	14.46	ns
	P (mmol/l)	1.50	1.45	1.45	1.40	ns
	Na (mmol/l)	40.00	43.40	44.20	45.00	ns
HCa	K (mmo/l)	6.00	6.43	6.54	5.80	ns
	Ca (mmol/l)	32.17	33.00	33.20	34.00	ns
	NO_3 (mmol/l)	14.00	15.30	13.47	13.89	ns
	P (mmol/l)	1.50	1.44	1.24	1.30	ns
	Na (mmol/l)	0.60	0.63	0.70	0.68	ns

The values are means (n=3). Statistical analysis refers to values that are within the same row, according to the Duncan's multiple comparison test: * indicate significance at $p< 0.05$; ns =not significant.
