# Supplementary figures and images for: Effects of NaCl and CaCl2 as Eustress Factors on Growth, Yield, and Mineral Composition of Hydroponically Grown Valerianella locusta
Source: Plants (Basel). 2023 Mar 26;12(7):1454. doi: 10.3390/plants12071454 (PMC10097257; doi:10.3390/plants12071454)

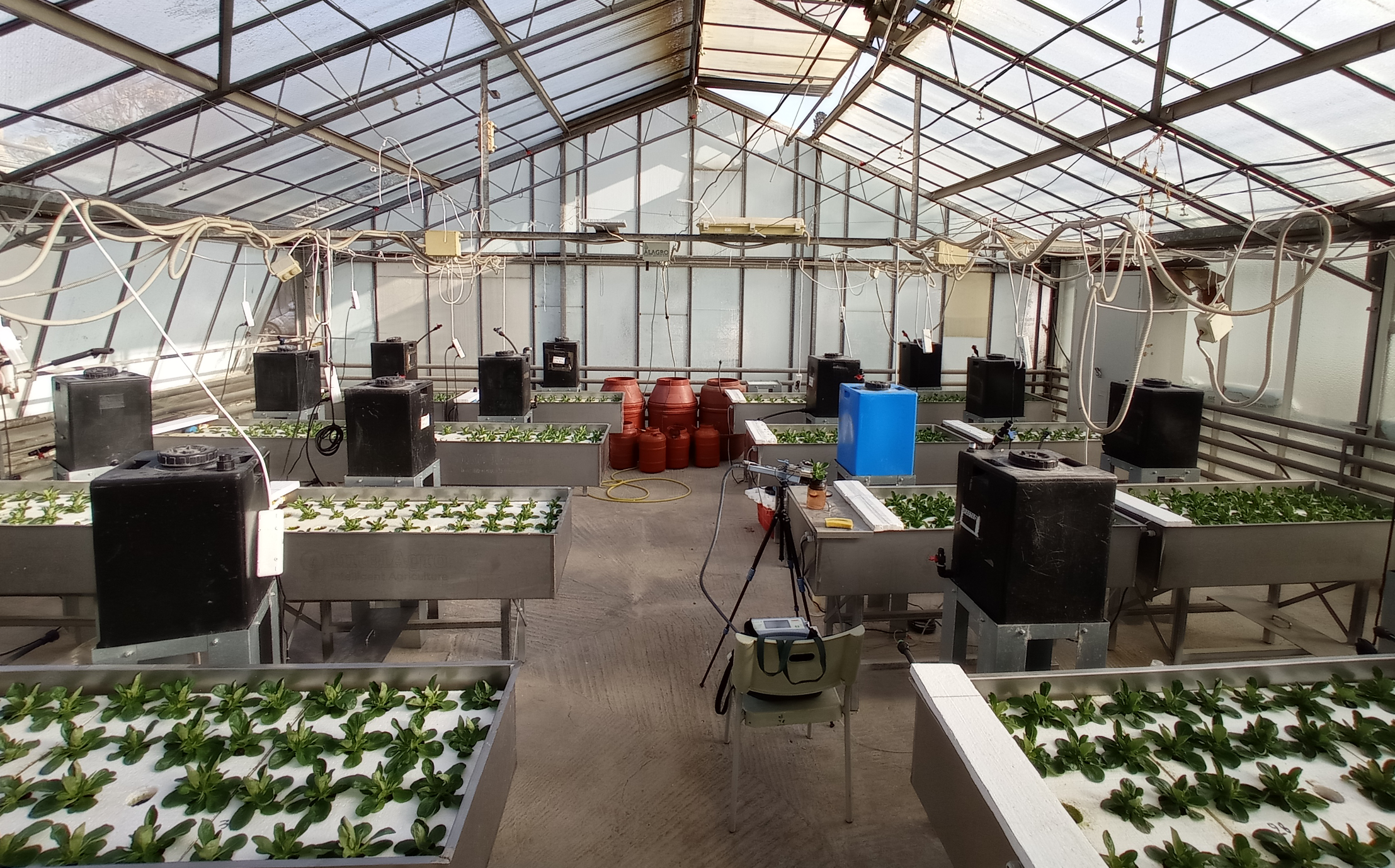

Supplement: Supplementary file 1 [file plants-12-01454-s001.zip › Figure S1.png]
